# Arthroscopic Subacromial Decompression vs. Physical Therapy for Stage II Shoulder Impingement Syndrome: A Comparative Review of Functional and Social Impact

**DOI:** 10.7759/cureus.96100

**Published:** 2025-11-04

**Authors:** Garrett J Rutt, Chloe Esch, Marcia Ballantyne

**Affiliations:** 1 Orthopaedic Surgery, Lake Erie College of Osteopathic Medicine, Bradenton, USA; 2 Physical Medicine and Rehabilitation, Lake Erie College of Osteopathic Medicine, Bradenton, USA; 3 Pathology, Lake Erie College of Osteopathic Medicine, Bradenton, USA

**Keywords:** arthroscopic shoulder surgery, arthroscopic subacromial decompression, neer's classification, patient centered care, shared decision making (sdm), shoulder impingement syndrome, social determinants of health (sdoh), subacromial decompression

## Abstract

Shoulder impingement syndrome is a highly frequent shoulder pathology that accounts for a large majority of visits in the primary care and orthopedic setting. Stage II impingement syndrome, defined as a progression of stage I impingement that occurs once the rotator cuff tendons have undergone fibrosis and tendonitis, is a critical phase in shoulder joint pathology that requires intervention. Although various treatments exist, including arthroscopic subacromial decompression (ASD) and physical therapy (PT), comparative data on their effectiveness, specifically for stage II impingement syndrome, remains limited. This review evaluates and compares the long-term outcomes of ASD and structured PT in specifically managing stage II shoulder impingement syndrome, focusing on pain relief, functional recovery, and return to daily activities with improvement in overall quality of life. Uniquely, this review also analyzes the social determinants of improved functional outcomes and the role that shared decision-making (SDM) plays in patient functionality and satisfaction. A comprehensive literature search was conducted using PubMed and Google Scholar, including only English-language, full-length clinical trials and randomized controlled trials that specifically addressed stage II shoulder impingement syndrome. Studies were screened using predefined inclusion and exclusion criteria, resulting in a final set of six articles for analysis. Outcomes were synthesized in a comparative table across both interventions. After analyzing multiple studies, ASD demonstrated no statistically significant benefit over PT in pain reduction, disability, work capability, muscle integrity, or patient-reported functional outcomes over two- to five-year follow-ups. Current evidence does not support the routine use of arthroscopic subacromial decompression over structured physical therapy for the management of stage II shoulder impingement syndrome, as both approaches yield comparable outcomes in pain reduction, functional improvement, and return to daily activities. Literature also supports improved outcomes with greater SDM preoperatively. These findings highlight the importance of adopting a personalized, patient-centered treatment strategy. In developing an optimal care plan, it is important to consider a range of individual factors, including the patient's age, occupation, access to emotional and physical support, financial circumstances, and long-term recovery goals.

## Introduction and background

Shoulder pain is a major issue in the world of healthcare, estimated to account for 7%-34% of all primary care and orthopedic office patient visits worldwide. Of those patient visits, it is believed that shoulder impingement syndrome is the underlying etiology in 44%-65% of those cases [1]. Generally speaking, shoulder impingement is a broad term that is given to any individual with pain upon overhead movement; however, a number of pathophysiological processes could account for this pain including shoulder instability, scapular dyskinesias, bicep tendinitis, superior labrum anterior to posterior (SLAP) tears, and chronic stiffness of the posterior capsule [2]. Ultimately, impingement is the result of compression of the rotator cuff muscles by superior structures (e.g., acromioclavicular joint, acromion, coracoacromial ligament) leading to inflammation and development of bursitis, regardless of the underlying etiology [3]. Neer outlined three different stages for classifying shoulder impingement [4]. Stage I is common in individuals under the age of 25 and is described as acute inflammation, edema, and hemorrhage of the rotator cuff conjoint tendon. Stage II impingement typically occurs in individuals 25-40 years of age and is a continuation of untreated stage 1 impingement, occurring once the rotator cuff tendon undergoes fibrosis and tendonitis. Stage III is common in patients over the age of 40 and occurs after the mechanical disruption of the rotator cuff tendon has occurred. During this stage, osteophyte formation will also occur along the coracoacromial arch [4]. Although multiple studies have demonstrated the benefits of arthroscopic subacromial decompression (ASD) in improving function and reducing pain for patients with stage II shoulder impingement syndrome [5,6], there is a lack of literature directly comparing the efficacy of ASD to physiotherapy or physical therapy (PT) specifically for this condition. Additionally, there is limited research to date on the impact of shared decision-making (SDM) on functional outcomes and patient satisfaction, particularly in the arthroscopic surgical setting. This review aims to fill these gaps by comparing the effectiveness of ASD versus PT in terms of pain relief, functional recovery, and return to daily activities, while also examining the impact of SDM. To our knowledge, this represents the first comprehensive, up-to-date review focusing specifically on stage II shoulder impingement syndrome that integrates both functional and social outcomes associated with SDM.

## Review

Methods

**Figure 1:**
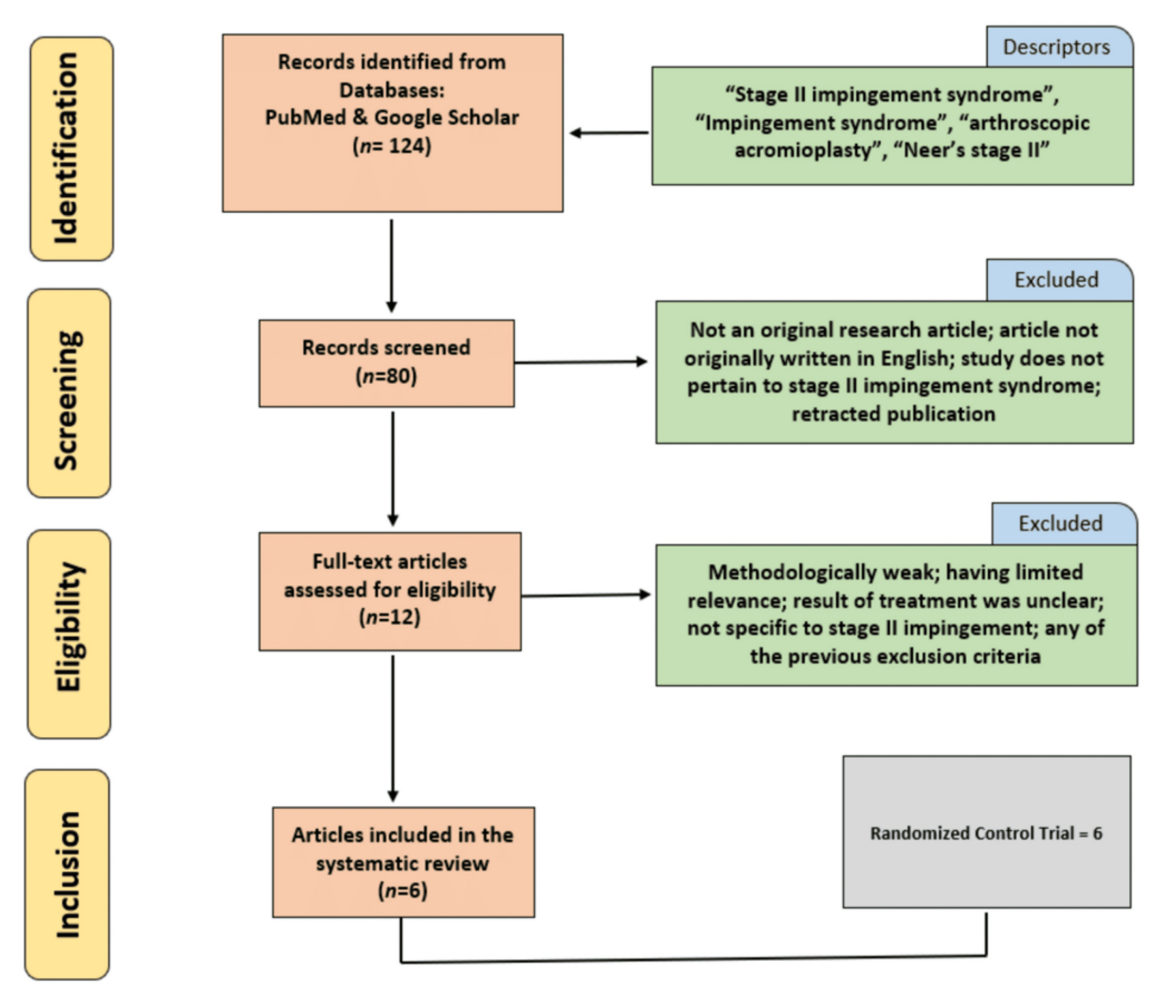
PRISMA Diagram Diagram showing the article search and selection process. PRISMA: Preferred Reporting Items for Systematic Reviews and Meta-Analyses.

This research project focusing on the long-term functional outcomes of performing arthroscopic subacromial decompression and structured physical therapy in treating patients with stage II impingement syndrome, as well as the social impact of shared decision-making (SDM), follows the Preferred Reporting Items for Systematic Reviews and Meta-Analyses (PRISMA) guidelines [7,8]. The review protocol was registered using International Prospective Systematic Review Registration Online (PROSPERO), protocol CRD420251145706 [9]. A flowchart of the article search and selection process, including reasons for exclusion, is shown below in Figure 1. To our knowledge, this represents the first comprehensive, up-to-date review focusing specifically on stage II shoulder impingement syndrome that integrates both functional and social outcomes associated with SDM. The primary literature for this review were randomized control trials collected from PubMed and Google Scholar databases. Only articles that clearly stated stage II impingement syndrome were included in the review. Keywords utilized to gather literature included “stage II impingement,” “Neer’s stage II,” “impingement,” and “arthroscopic acromioplasty.”

Inclusion Criteria

Only full-length clinical trials, randomized control trials, and reports originally published in the English language from the years 2006 to 2025 were included in the review.

Exclusion Criteria

The following exclusion criteria were considered: studies that did not clearly state stage II impingement syndrome, unpublished results, unclear results, retracted publications, and non-English papers.

Data Synthesis

Initially, a total of 124 articles were screened. After investigation of the literature and application of the exclusion criteria, a total of six different articles were included in the review. Articles published between the years 2006 and 2025 were included if they met the specified inclusion criteria listed above. The last search was performed in August 2025. Data was then documented from each article and subsequently merged to create a table comparing various outcome measures between the arthroscopic subacromial decompression and physical therapy groups.

Review Process

The article selection process involved precise and periodic review by all authors, following the existing inclusion and exclusion criteria. Each stage throughout the review and data synthesis process was double checked by all authors. In the case of unclear findings or disagreements among authors, the entire research team discussed and came to a consensus.

Risk of Bias

Due to the limited number of studies and the vast differences in methodology among the studies included, a formal meta-analysis or bias scoring assessment tool was not applied.

Results

A randomized controlled trial was performed to evaluate the two- and five-year follow-up outcomes of arthroscopic acromioplasty followed by a structured exercise program compared to a structured exercise program alone [10]. The study included 140 participants aged 18 to 60 years, all diagnosed with stage II shoulder impingement syndrome. Prior to enrollment, MRI imaging was used to exclude other intra-articular pathologies. The primary outcome was self-reported shoulder pain, measured using a 0-10 Visual Analog Scale (VAS) [11], where scores of 0-3 indicated minimal or no pain. Secondary outcomes included disability, working ability, night pain (VAS), Shoulder Disability Questionnaire (SDQ) score [12], and the number of painful days reported in the three months prior to each follow-up. In the two-year follow-up [13], there was no statistically significant difference in self-reported pain between the acromioplasty group (mean VAS: 2.5) and the exercise-only group (mean VAS: 2.9; p = 0.37). Similarly, no significant differences were observed in secondary outcomes: disability (2.0 vs. 2.6; p = 0.21), working ability (both 8.0; p = 0.96), night pain (2.0 vs. 2.6; p = 0.19), SDQ score (24.2 vs. 32.8; p = 0.13), and number of painful days in the previous three months (13.9 vs. 19.7; p = 0.22). At five years [14], 31 patients were lost to follow-up, the results remained consistent. No statistically significant difference was found in self-reported pain between the acromioplasty group (mean VAS: 1.9) and the exercise-only group (mean VAS: 2.2; p = 0.44). Secondary outcomes also showed no significant differences: disability (1.5 vs. 1.8; p = 0.57), working ability (7.8 vs. 7.5; p = 0.41), night pain (both 1.7; p = 0.95), SDQ score (16.9 vs. 22.2; p = 0.33), and painful days in the previous three months (12.2 vs. 11.8; p = 0.94). In conclusion, both the two- and five-year follow-up data demonstrate no statistically significant benefit of arthroscopic acromioplasty when added to a structured exercise program compared to exercise alone, across all measured outcomes, as shown in Table 1.

**Table 1:** Outcomes at Two- and Five-Year Follow-up Mean number of days patients experienced each outcome at two- and five-year follow-ups. SDQ: Shoulder Disability Questionnaire.

	Surgery Two Year	PT Two Year	p-value (Two Year)	Surgery Five Year	PT Five Year	p-value (Five Year)
Self-reported pain	2.5	2.9	0.37	1.9	2.2	0.44
Disability	2.0	2.6	0.19	1.5	1.8	0.57
Working ability	8.0	8.0	0.96	7.8	7.5	0.41
Night pain	2.0	2.6	0.19	1.7	1.7	0.95
SDQ score	24.2	32.8	0.13	16.9	22.2	0.33
Painful days in prior three months	13.9	19.7	0.22	12.2	11.8	0.94

A similar study was performed as a randomized controlled trial to evaluate the five-year effects of arthroscopic acromioplasty followed by a structured exercise program versus a structured exercise program alone on the integrity of rotator cuff muscles [15]. Specifically, the study assessed muscle volume (supraspinatus, infraspinatus, and subscapularis), fatty degeneration, and full-thickness tears of the supraspinatus tendon. The trial included 140 patients aged 23-60 years with confirmed stage II shoulder impingement syndrome and no additional joint pathology on MRI. At the five-year follow-up, no statistically significant differences were observed between the two groups in muscle volume: supraspinatus (p = 0.6), infraspinatus (p = 0.9), and subscapularis (p = 0.5), with a significance threshold of p < 0.05. In terms of fatty degeneration, 65% of patients in the acromioplasty group showed signs of fatty streaks in the rotator cuff muscles compared to 54% in the exercise-only group; however, this difference was not statistically significant (p = 0.3). Additionally, 15 patients were found to have a supraspinatus tendon tear at the five-year follow-up, eight of whom had undergone surgery. Overall, the study concluded that arthroscopic acromioplasty did not result in significant differences in muscle volume, fatty degeneration, or incidence of tendon tears when compared to structured exercise alone.

Haahr and Andersen conducted a randomized controlled trial to assess long-term four to eight years’ socioeconomic and functional outcomes following arthroscopic subacromial decompression versus physiotherapy and exercise in patients with stage II subacromial impingement syndrome [16]. A total of 90 adults, aged 18-55 years, with confirmed stage II impingement were enrolled; however, 11 participants were lost at different points throughout the study and only 79 participants completed the final follow-up questionnaire. Primary outcome measures included the marginalization index (defined as the proportion of a 52-week period during which any form of public income transfer was received), the sick leave index, and the disability pension index, assessed four years post-intervention. Secondary outcomes included self-reported employment status, work capability (on a 0-10 scale), global perceived change (“completely recovered,” “much improved,” “improved,” “unchanged,” “worse,” or “much worse”), and shoulder function, evaluated using Patient-Reported Impairment Measure (PRIM) scores between years four and eight. No statistically significant differences were observed between the arthroscopic subacromial decompression and physiotherapy groups with respect to employment status (20 vs. 21 participants employed), self-rated work capability (mean score: 5.3 vs. 5.0), or PRIM scores (mean: 25.8 vs. 25.8). Similarly, global perceived improvement did not differ between groups. Both cohorts exhibited comparable increases in the disability pension index relative to baseline, with no significant difference at the four-year follow-up (0.25 vs. 0.21). While the marginalization index was significantly elevated in the arthroscopic subacromial decompression group at one year post-intervention (0.45 vs. 0.25), this difference was not sustained at four years (0.20 vs. 0.24; p > 0.05). Overall, the findings demonstrated no clinically or statistically significant long-term advantage of arthroscopic subacromial decompression over structured physiotherapy and exercise in terms of socioeconomic reintegration, work capacity, or patient-reported functional outcomes, as evidenced in Table 2.

**Table 2:** Socioeconomic Reintegration, Work Capacity, or Patient-Reported Functional Outcomes Mean marginalization index, sick leave index, and disability pension index from two years before until four years after intervention. Also shows self-reported outcomes of average work capability and PRIM scores (global perceived change and shoulder function), as well as the number of participants with employment in years four to eight after intervention. PRIM: Patient-Reported Impairment Measure.

	Marginalization Index (n=mean)	Sick Leave Index	Disability Pension Index (n=mean)	Employment Status (n=quantity)	Work Capability (n=mean)	Global Change and Function (n=mean PRIM)
Surgery	0.20	-0.11	0.25	20	5.3	25.8
Physiotherapy	0.24	-0.06	0.21	21	5.0	25.8

The findings from the studies discussed above suggest that an additional significant factor may be influencing the functional outcomes observed. One observational, longitudinal survey-based study investigated whether shared decision-making (SDM) contributes to improved patient satisfaction and self-reported functional outcomes in a cohort of approximately 2,779 individuals undergoing total joint arthroplasty (1,704 knee and 1,075 hip replacements) [17]. Participants were surveyed both preoperatively and one year postoperatively using several instruments, including the Patient-Reported Outcome Measures (PROMs) survey for patient experience, the three-item CollaboRATE tool for assessing SDM, and the Oxford Knee or Hip Score for functional assessment. The study found a moderate, positive correlation between preoperative CollaboRATE scores and Oxford scores at the 12-month follow-up, even after adjusting for confounding variables (p = 0.01). Additionally, patients reported significantly higher satisfaction with their care (p = 0.003) and were more likely to recommend their surgery to others (p = 0.005). Given the limited research on SDM in the setting of arthroscopic shoulder surgery, this study examining SDM in total joint arthroplasty of the knee and hip was included. The findings suggest that higher levels of SDM prior to surgery are associated with improved postoperative functional outcomes and enhanced patient experience.

Discussion

To our knowledge, this is the first review to incorporate both functional and social outcomes in the comparison of ASD and PT for stage II impingement. This review highlights that arthroscopic subacromial decompression may not be the most effective treatment for all patients with stage II impingement syndrome as there were no statistically significant differences in the major outcomes tracked throughout the studies included in this review, mainly self-reported pain and functional usage. A comparative analysis of multiple studies examining surgical intervention versus physical therapy reveals no statistically significant difference in long-term outcomes, particularly self-reported pain relief, among patients treated for this specific condition [5-6,10,13-16].

These findings suggest that a patient-centered, shared decision-making approach may lead to improved long-term outcomes. As mentioned above, evidence indicates that patients who report a greater degree of involvement in preoperative decision-making experience better functional results and overall satisfaction with their care [17].

Given these insights, a range of external factors should be carefully considered when determining the most appropriate treatment. Variables such as the patient's age, occupation, financial considerations, and post-intervention support systems can significantly influence recovery. For example, the ideal treatment for a college athlete may differ substantially from that of an older adult. Supporting this, one of the previously mentioned studies found that individuals who lived alone or had demanding work obligations reported higher levels of pain at both two- and five-year follow-ups, regardless of treatment type [10]. These findings underscore the importance of evaluating the patient holistically and incorporating shared decision-making into the clinical process.

In addition to the benefits of shared decision-making, evidence also supports the importance of patient trust and adherence to the initial treatment plan. While patients naturally desire a quick return to full health and preinjury level of activity, this urgency can sometimes lead to premature abandonment of the primary intervention and delayed recovery. A prior research study found that patients who changed treatment strategies before completing their initial plan experienced higher levels of self-reported pain, disability, pain at night, SDQ score, and a decreased working ability compared to those who remained within their assigned treatment group [10]. These findings emphasize the need for both collaborative decision-making and patient commitment to the agreed-upon plan in order to optimize recovery and long-term success.

Although current studies consistently suggest no significant difference in long-term outcomes between surgical and non-surgical treatments for stage II impingement syndrome, several limitations remain. Future research should aim to control for external factors, such as marital status, occupational demands, and patient-specific treatment goals, that may influence outcomes. While focusing this review on stage II impingement syndrome specifically keeps the focus narrow, it also limits the generalizability of these findings and more future studies are needed to strengthen the significance of these findings. Moreover, stratifying patients into narrower age ranges while maintaining randomization could help minimize confounding variables, as younger individuals may experience faster recovery and inadvertently skew results.

## Conclusions

This review set out to examine the long-term functional and social outcomes in patients with stage II impingement syndrome treated either with arthroscopic subacromial decompression or structured physical therapy, with a primary focus on self-reported pain. After evaluating multiple long-term randomized controlled trials, evidence indicates no statistically significant differences between the two treatment approaches. Findings also highlight the importance of considering social factors, such as marital status and occupational demands, and suggest that increased shared decision-making in the preoperative period is associated with improved postoperative functional outcomes and patient satisfaction.

These findings suggest that, while the clinical efficacy of these treatments is comparable, other factors need to be considered to improve long-term outcomes. Rather than comparing approaches, treatment should be tailored to a more individualized approach, meeting the patient’s personal goals and preferences. The role of SDM allows physicians to better understand their patients’ values, improving both functional and social outcomes for all patients.

Future research should look beyond MRI findings and physician preference to consider social determinants of health, such as support systems, age, occupation, financial resources, and personal treatment goals. Addressing these factors can improve both symptom resolution and overall quality of life. Ultimately, these findings emphasize the need for a more personalized, patient-centered approach when treating stage II shoulder impingement syndrome. Treatment should reflect not just imaging results, but the unique circumstances and values of each patient. The implementation of SDM supports this holistic approach to treatment, improving both functional and social outcomes.
